# Canine preclinical safety evaluation of a multimodal nanoparticle agent (Nanotrast-CF800) for image-guided cancer surgery

**DOI:** 10.1371/journal.pone.0296913

**Published:** 2024-06-13

**Authors:** Jennifer Wan, Andrea Sanchez, Charly McKenna, Stephanie Nykamp, Michelle L. Oblak

**Affiliations:** 1 Department of Clinical Studies, Ontario Veterinary College, University of Guelph, Guelph, Ontario, Canada; 2 Department of Biomedical Sciences, Ontario Veterinary College, University of Guelph, Guelph, Ontario, Canada; University of Porto, PORTUGAL

## Abstract

Surgical oncology often requires the use of contrast-enhanced cross-sectional imaging preoperatively to characterize solitary tumours and identify sentinel lymph nodes. Intraoperative optical guidance can effectively aid tissue-sparing tumour excision and locate sentinel lymph nodes. Nanotrast-CF800 (CF800) is a novel dual-modality contrast agent, which co-encapsulates iohexol and indocyanine green (ICG) within a liposomal nanoparticle. It was developed for preoperative and intraoperative imaging of solitary tumours and sentinel lymph node mapping and its efficacy has been demonstrated in preclinical animal models (Zheng et al. 2015). The objective of this study is to evaluate the safety profile of CF800 following intravenous administration in healthy dogs. Six research dogs were randomized into two groups. Group 1 received a low dose (1 mL/kg) and group 2 received a high dose (5 mL/kg). Dogs were placed under general anesthesia and a continuous rate infusion of CF800 was administered based on group allocation. Physiologic parameters including heart rate, respiratory rate, direct arterial blood pressure, cardiac output, and temperature were measured at set time points. Plasma concentrations of iohexol, ICG, and histamine were measured at set time points. Dogs underwent whole body computed tomography scans pre-injection, 2-, and 7-days post-injection (p.i.). Contrast enhancement was measured in select organ systems and great vessels at each time point. There were no significant changes in physiologic parameters following IV infusion of CF800 in all dogs. Plasma iohexol and ICG concentrations peaked at 1 day p.i., while histamine concentrations peaked at 30 minutes p.i. Significant contrast enhancement was noted within the liver, heart, aorta, and caudal vena cava on day 2 p.i., which was significantly different compared to baseline. Prolonged contrast retention within the liver was identified. Intravenous administration of CF800 was safe to use in healthy dogs with no significant systemic adverse effects.

## Introduction

Surgery is the primary modality for the treatment of solitary tumours and can have a significant impact on patient survival. Surgical oncology often requires the use of contrast enhanced CT prior to surgery to characterize solitary tumours and determine their size, location, and extent of disease [1–3]. This technique is particularly advantageous in areas involving critical anatomical structures, such as, the head and neck, to determine tumour resectability [1–3]. Computed tomography can be used to assess regional lymph nodes; however, may not be reliable in detecting metastatic lymph nodes [4]. Computed tomography lymphography is performed preoperatively to identify the sentinel lymph node (SLN) with a reported SLN detection rate ranging between 60–93% [5–7]. The ability to improve tumour visualization and determine SLNs using cross-sectional imaging can influence surgical performance and may have a positive effect on patient outcome.

Intraoperative optical imaging can effectively guide the surgeon to perform appropriate tissue-conserving surgeries for tumour excision, which is highly desirable to minimize patient morbidity while achieving tumour-free margins. The use of near-infrared fluorescence (NIRF) imaging and indocyanine green (ICG) has been shown to improve detection of tumour margins and identify metastatic lymph nodes [8–11]. Mehta *et al*. demonstrated that NIRF imaging of pulmonary tumours resulted in increased surgical margins between the tumour and remaining lung that were necessary to achieve clean margins, compared to surgeon prediction of surgical margins [10]. This finding suggests that precise tumour margins can be determined with the use of intraoperative optical imaging techniques in order to obtain tumour-free margins and reduce excessive excision of normal tissue parenchyma.

A potential limitation associated with iohexol is its short half-life, which allows it to become rapidly distributed and excreted [5, 11]. Therefore, if repeated preoperative CT imaging will be performed, then multiple administrations of iohexol would be required. In addition, timing of injection of fluorescent contrast agents may require additional hospital visits or prolonged hospitalization. A novel liposomal nanoparticle dual-modality contrast agent, Nanotrast-CF800, was developed to overcome these challenges [12]. CF800 can be utilized for the preoperative and intraoperative visualization of tumours and SLN mapping by co-encapsulating both iohexol and ICG within a liposomal nanoparticle. Iohexol is a water-soluble based nonionic contrast agent commonly used for CT imaging, and ICG is an FDA-approved fluorescent contrast agent. Both contrast agents are commercially available, clinically approved, and are associated with minimal adverse reactions in both human and veterinary patients [11, 13]. Following intravenous injection, nanoparticles have been found to be retained and accumulate in tumours and metastatic lesions through the enhanced permeability and retention (EPR) effect [14]. This effect occurs as result of increased tumour vascular permeability due to poorly aligned endothelial cells and wide endothelial gaps, and lack of lymphatic drainage [14]. In a study assessing CF800 for image-guided head and neck surgery in rabbits, peak fluorescence of the primary tumour was noted at 96 hours post-administration [15].

The liposome capsule is intended to prolong the biological half-life of the encapsulated contrast agents, which can allow for repeated preoperative CT imaging with the adjunctive use of intraoperative NIRF imaging via a single preoperative injection [12]. In a preclinical animal model, tumours and metastatic lymph nodes could be identified by CT contrast enhancement and intraoperative fluorescence in tumour-bearing mice and rabbits up to 4 days post-injection (p.i.) [12]. The authors of that study proposed that the use of this dual modality contrast agent could allow for appropriate presurgical planning, as well as, guide precise tumour resection and SLN identification. This can overall improve patient outcome by reducing the risk of tumour recurrence and the need for repeated surgical procedures.

In veterinary medicine, challenges associated with evaluation of local and metastatic disease results in wider surgical margins, which can increase surgical morbidity. The identification and removal of SLNs is also a growing field of interest. The authors have conducted a pilot study demonstrating the efficacy of CF800 for SLN mapping following local administration within the oral cavity of healthy dogs [6]. The intravenous administration of CF800 may allow for more accurate surgical planning both preoperatively and postoperatively through contrast-enhancement and NIRF, respectively, of primary and metastatic lesions. As with humans, veterinary patients would benefit greatly from access to advanced methods for diagnosis and intraoperative tumour bed evaluation, resulting in less aggressive surgery and improved disease-free and survival outcomes.

In the authors’ experience, surgery is often scheduled at a later date from advanced imaging. The administration of CF800 at the time of the initial surgical consultation may result in prolonged retention of the contrast agent within tissues, which would allow for advanced imaging then surgery to be performed over a certain time period. Re-administration of the contrast agent and further delay of surgical treatment can potentially be avoided.

The purpose of this study is to perform an initial safety evaluation in purpose-bred research dogs following an intravenous infusion of Nanotrast-CF800. Following this safety study, a clinical trial will be developed to evaluate the efficacy of this novel nanoparticle agent in tumour-bearing dogs. The results obtained from these studies will provide valuable translational information that will impact the human population. We hypothesize that an intravenous infusion of Nanotrast-CF800 will not lead to significant adverse systemic events. Secondarily, we hypothesize that an intravenous infusion of Nanotrast-CF800 will result in prolonged CT contrast enhancement of tissues of interest.

## Materials and methods

### Animals

Six healthy purpose-bred adult research dogs obtained from a research colony (Intervivo Solutions, Fergus, Ontario, Canada) were utilized for the study. This study was approved by the University of Guelph Animal Care Committee (AUP3534). Dogs were determined to be healthy and free of disease based on physical examination, a complete blood count, biochemistry profile and urinalysis. At 48 hours prior to the start of the study, dogs were administered dexamethasone 0.1 mg/kg IM and diphenhydramine 2 mg/kg IM to reduce the risk of an anaphylactic reaction. Dogs were randomized into two groups using a random number generator to receive either a low dose (1 mL/kg, Group 1) or high dose (5 mL/kg, Group 2) of CF800. A complete blood count, serum biochemical profile, and urinalysis were performed for all dogs prior to the injection and on days 2 and 7 p.i.. Dogs were monitored for 10 days following CF800 infusion. At the completion of the study, dogs were returned to the research colony.

### CT imaging

All dogs underwent a whole-body CT scan 24 hours prior to the start of the study (day -1). The dogs were sedated using butorphanol 0.1–0.3 mg/kg IV and dexmedetomidine 2–5 ug/kg IV. Dogs were positioned in dorsal recumbency and CT imaging was performed using a 16-slice detector CT (GE Brightspeed CT scanner, GE Healthcare, Milwaukee, Wisconsin, United States) with data collected using 0.625mm slice thickness and standardized protocol in helical mode, 0.8 second rotation time, collimator pitch of 1, 120 kV and 200mAs. Dogs then received an intramuscular injection of atipamezole at a volume equivalent to the amount of dexmedetomidine previously administered and returned to their housing unit. Whole body CT scans were repeated on days 2 and 7 p.i. using the previous sedation protocol.

### Nanotrast-CF800 infusion

On the day of the study (day 0), baseline physical examination and vital parameters (heart rate, respiratory rate, rectal temperature, and indirect oscillometric blood pressure) were obtained. A 20 – 22-gauge intravenous catheter was placed in the right or left cephalic vein. Two milliliters of whole blood were collected for baseline measurements of iohexol, ICG, and histamine levels. A test dose of CF800 was administered slowly at 0.1 mL/kg IV. Vital parameters were repeated every 5 minutes for a total of 15 minutes and any changes were recorded. An additional 2 mL of venous whole blood was collected 5 minutes following the test dose injection for iohexol and ICG analysis. Dogs were then premedicated using hydromorphone 0.05 mg/kg IV. General anesthesia was induced using propofol 1–4 mg/kg IV titrated to effect. Dogs were intubated using a cuffed endotracheal tube and general anesthesia was maintained using isoflurane delivered on 100% oxygen with an end-tidal isoflurane concentration (ETISO%) of 1.3%. Following anesthetic induction, a 16-gauge indwelling double lumen jugular catheter (MILA International Inc., Brampton, Ontario, CA) was placed in either the right or left jugular vein and bandaged in place. An arterial catheter was placed using a 22-gauge needle in the left or right dorsal pedal artery. Intermittent positive pressure ventilation was provided at a rate of 8–10 breaths per minute and tidal volume (TV) of 10-15mL/kg to maintain an end-tidal CO_2_ of 35 to 45 mmHg. Dogs received intravenous isotonic crystalloid fluid therapy (Plasmalyte) at a rate of 5mL/kg/hr.

Nanotrast-CF800 was infused at an initial rate of 5 mL/hr. The rate doubled every 15 minutes until infusion of the total volume was completed (determined based on group allocation). Group 1 dogs completed the infusion in 45 minutes and group 2 dogs completed the infusion in 75 minutes. A multiparameter monitor (Datex-Ohmeda S/5 Anesthesia Monitor; GE Healthcare, Helsinki, Finland) was used to record heart rate and rhythm (ECG), respiratory rate, end-tidal carbon dioxide (ETCO_2_), ETISO%, esophageal temperature, peripheral pulse oximetry (SpO_2_), indirect blood pressure, systolic (SAP), diastolic (DAP), and mean (MAP) arterial pressures, tidal volume, and peak inspiratory pressures. Dogs were monitored using an electrocardiogram, direct arterial blood pressure, pulse oximeter, and temperature. Tachycardia was defined as a heart rate > 160 bpm and bradycardia was defined as a heart rate < 50 bpm. Hypotension was defined as a mean arterial pressure of < 60 mmHg and/or systolic pressure of < 90 mmHg. Cardiac output was measured every 15 minutes using a lithium dilution cardiac output monitoring system (LiDCO Ltd, Lake Villa, IL, USA) and was set up and performed as previously described [16]. A bolus dose of 0.008mmol/kg of lithium chloride was administered into the jugular catheter to re-calibrate the LiDCO system following each cardiac output measurement. Other cardiorespiratory and physiological parameters were recorded every 5 minutes up to 30 minutes after the completion of the infusion. In addition, 2 mL of venous whole blood was collected from the jugular catheter every 15 minutes for histamine analysis. Blood samples for histamine analysis were placed in heparinized tubes and immediately spun in a refrigerated centrifuge at 3000rpm at 4°C. For each sample, plasma was separated and placed in cryotubes in 0.5 mL aliquots. The cryotubes were stored in a freezer at -80°C until assay. Following completion of the study, dogs were recovered from anesthesia. The arterial catheters were removed, and dogs were returned to their housing unit.

### Plasma iohexol and ICG analysis

Daily physical examinations were performed on all dogs for a total of 9–10 days post-infusion. Two milliliters of venous whole blood were collected from the indwelling jugular catheter daily. Blood samples were placed in heparinized tubes and immediately spun in a refrigerated centrifuge at 3000rpm at 4°C. For each sample, plasma was separated and placed in cryotubes in 0.5 mL aliquots. The cryotubes were stored in a freezer at -80°C until assay. The samples were then shipped to an external lab for measurement of plasma iohexol and ICG concentrations.

### Histamine concentration assay

Histamine concentrations were measured from collected plasma samples using a commercialized enzyme immunoassay (EIA) Histamine Kit (Immunotech s.r.o, Prague, Czech Republic) according to the manufacturer’s recommendations. The assay has an analytical sensitivity of 0.057 ng/mL. Briefly, an acylation reagent, conjugate solution, wash solution, and substrate solution were prepared. Plasma samples were thawed at room temperature and 200 uL of plasma from each sample were placed into separated centrifuge tubes then mixed with 50 uL of acylated reagent. Using a 96 well plate, the provided control sample and calibrated samples (0 nM, 1.3 nM, 3.6 nM, 12 nM, 35 nM, and 100 nM) were placed into individual wells followed by the plasma solution. Plasma samples were performed in triplicates. Conjugate solution was added to each well then, the samples incubated for 2 hours at 2–8°C with shaking (350 rpm). Following incubation, the plate was washed with the wash solution three times. An additional 200 uL of conjugate was added to each well and the samples incubated for 30 minutes at room temperature with shaking in the dark. A stop solution was then added to each well. The plate was run through a microplate reader (Epoch 1310285, V. 2.04.11, BioTek Instruments Inc, Winooski, VT, USA) at a wavelength of 405 nM and the results displayed in an Excel worksheet (Microsoft Excel 2002, Microsoft Corporation, Washington, USA)

For three samples, the resultant concentrations exceeded 100 nM, thus, further dilutions were performed to obtain an absolute number. This was performed by diluting the plasma sample in bovine serum albumin solution (40mg/ml; Sigma-Aldrich Co, St Louis, MO) to create a 1:10 dilution as per the procedural instructions provided with the histamine kit. The sample was then prepared as previously described and run through the microplate reader. Results were provided in nM (nmol/L) units, which was converted to ng/mL by dividing nmol/L units by 9.

### Image analysis

All CT images were analyzed using AGFA Enterprise Imaging Platform (Agfa Healthcare N.V, Mortsel, Belgium). Evaluation of the liver, spleen, heart, aorta, and caudal vena cava were performed at each time point in a soft tissue window reconstruction (WW:400 WL:40) and axial plane. For evaluation of the liver, a region-of-interest (ROI) measuring 1.5cm^2^ was manually drawn in each hepatic division (left, central, and right) within the parenchyma in a region that was devoid of blood vessels or focal lesions. Measurements were performed in 3 separate slices minimizing variation in ROI placement (total of 9 measurements per scan). Evaluation of the spleen was performed similarly to the liver but utilizing two ROIs (total of 6 measurements per scan). Measurements were performed based on a previous study.^11^ Evaluation of the heart was performed using a 1.5cm^2^ ROI within the left ventricle taking care to avoid the ventricular wall. The aorta and caudal vena cava were evaluated with an ROI of 0.5cm^2^ at the level of the heart and diaphragm, respectively. Data recorded included the minimum, maximum, mean, and standard deviation measured in Hounsfield Units (HU).

The mean contrast enhancement for each organ at each time point was determined. The percentage of contrast enhancement within each organ compared to the pre-injection image was calculated by using the following formula: [13]

$$\%\;Contrast\;Enhancement=\frac{Postcontrast\;density-Precontrast\;density}{Precontrast\;density\;}\;x\;100$$

### Statistical analysis

Continuous descriptive data was used to calculate mean +/- SD for normally distributed data. Shapiro-Wilk, Kolmogorov-Smirnov, Cramer-von Mises, and Anderson-Darling tests were used to assess for normality. A logit transform was performed on data that were not normally distributed. For percentage data, logit transform was also performed to meet the assumptions of normality. Analysis of variance (ANOVA) for repeated measures was used to determine any differences in mean iohexol, ICG, and histamine concentrations between groups and over time. For statistical comparison, only time intervals which had measured values for both groups were included. ANOVA for repeated measures was used to determine any differences in mean contrast enhancement between organ systems at each time point and between groups. Tukey and Dunnetts adjustments were performed for pairwise comparisons. Percent contrast enhancement between groups and over time was also determined using ANOVA for repeated measures. A general mixed linear regression model was used to evaluate for an association between histamine concentration and mean arterial pressure and cardiac output between groups and over time.

## Results

### General

Six healthy purpose-bred research dogs were utilized for this study. All dogs were intact female beagles. The mean body weight was 9.78 kg (range 8.9–11.3kg) and the median age was 2 years. All dogs completed the study and received the total dose based on group allocation. During administration of the IV test dose of CF800, all dogs exhibited mild to moderate sedation and hypersalivation, which spontaneously resolved within minutes. There were no changes in vital parameters (heart rate, respiratory rate, indirect blood pressure, and body temperature).

### Plasma iohexol, ICG, and histamine concentrations

**Fig 1:**
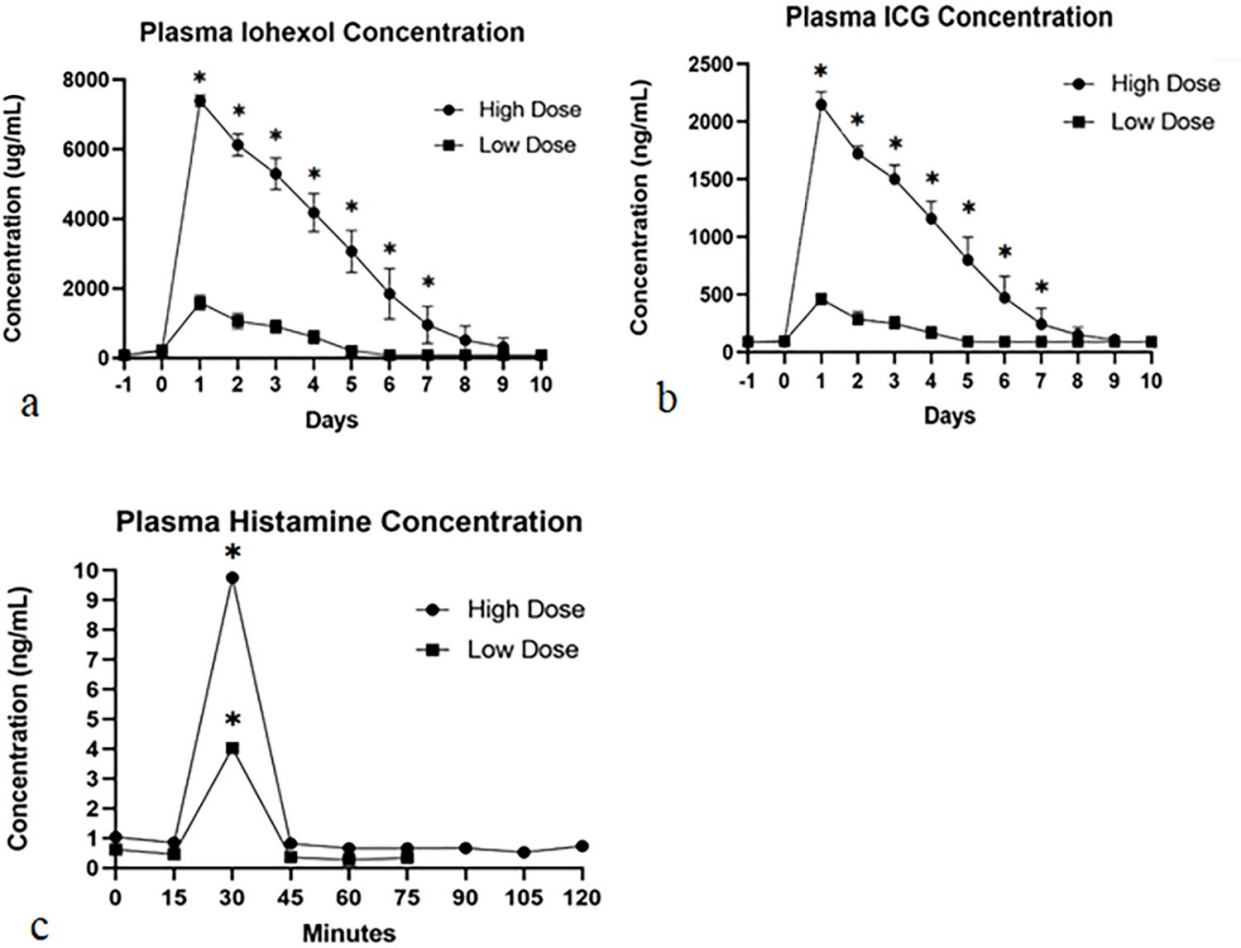
Graphical representation of plasma iohexol (a), ICG (b), and histamine (c) concentrations over time and between groups. Group 1 (■) Group 2 (●). * indicates significance (p<0.0001) between groups on Day 1 post-injection (a, b) and compared to baseline at 30 minutes post-injection (c).

The overall mean plasma iohexol, ICG, and histamine concentrations were determined for each group and at each time point (Fig 1). The mean iohexol and ICG concentrations significantly differed between groups and over time (p<0.0001). The mean iohexol concentration peaked at day 1 p.i. and was significantly higher in group 2 (7394.04 μg/mL; range 7057.22–7730.85 μg/mL) compared to group 1 (1596.75 μg/mL; range 1259.91–1933.54 μg/mL) (p<0.0001). Similarly, the mean ICG concentration peaked at day 1 p.i. and was significantly higher in group 2 (2147.06 ng/mL; range 2053.28–2240.85 ng/mL) compared to group 1 (459.68 ng/mL; range 365.90–553.46 ng/mL) (p<0.0001). Plasma histamine concentrations peaked at 30 minutes p.i. in both groups (Group 1 = 2.12 ng/mL, 95% CI [1.11,4.04 ng/mL], Group 2 = 9.42 ng/mL; 95% CI, [4.93,18.01 ng/mL]), which was significantly increased compared to baseline and between groups (p < 0.0001).

**Fig 2:**
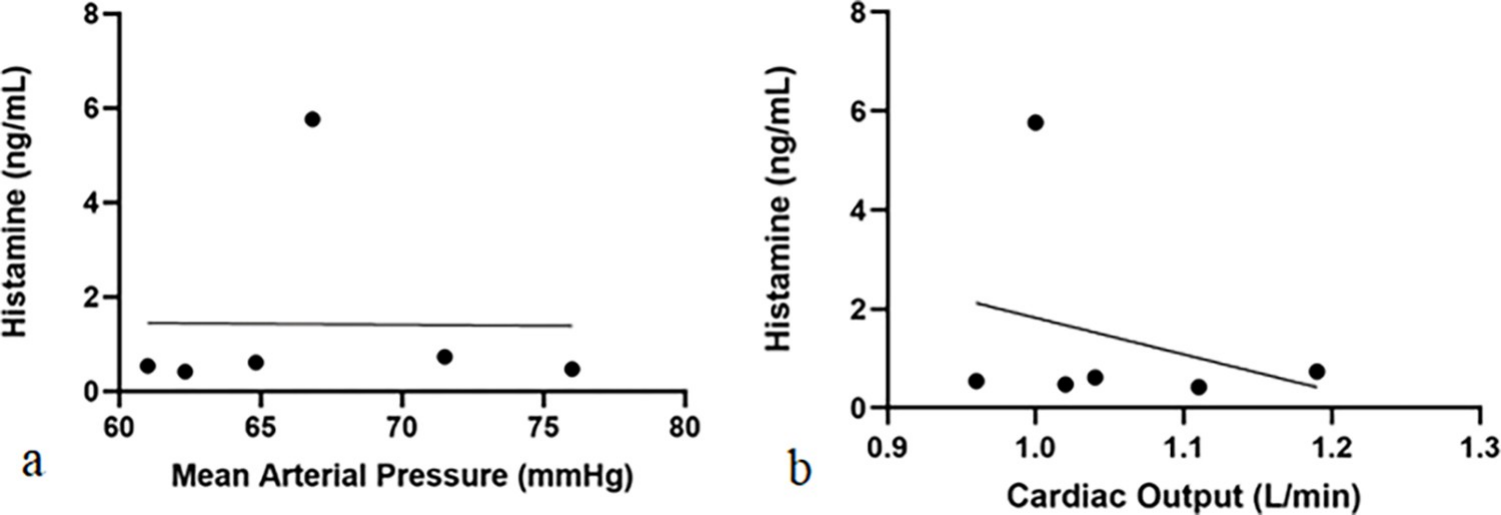
Correlation between overall histamine concentration and mean arterial pressure (a) and cardiac output (b) at 0, 15, 30, 45, 60, and 75 minutes.

A general mixed linear regression model was used to determine the overall relationship between plasma histamine concentration with cardiac output and mean arterial pressure. There was no association between histamine and cardiac output identified (slope = 0.986; p = 0.06). However, there was a positive association between histamine and mean arterial pressure and this was significant (p = 0.0009). For every logarithmic unit increase in histamine, mean arterial pressure also increased (slope = 0.028) (Fig 2).

### CT image analysis

**Fig 3:**
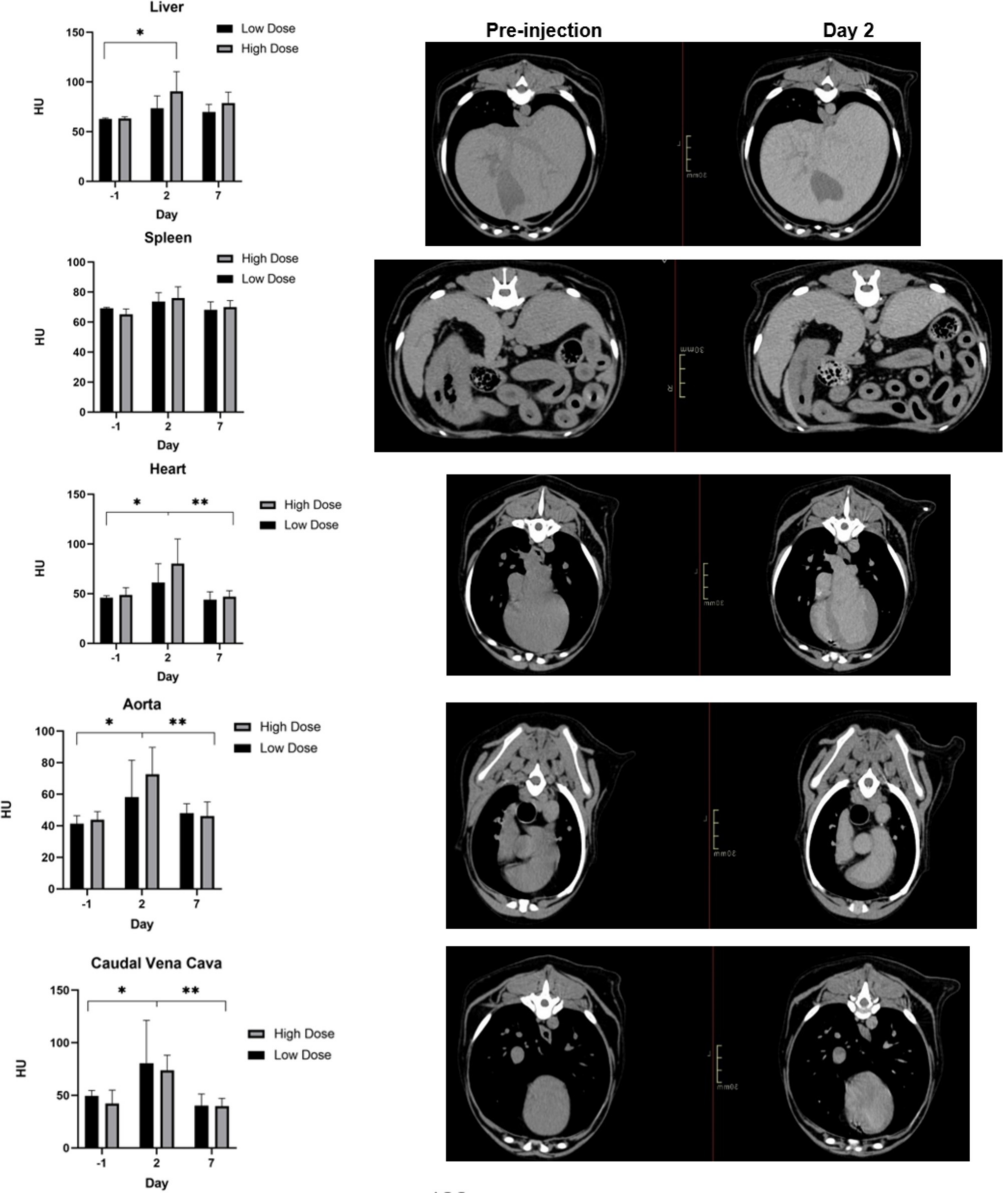
Mean contrast enhancement within select organs over time and between groups (left). CT images in the axial plane and soft tissue window (WW:400; WL:40) at day 2 post-injection from the same dog in group 2 (right). * indicates significance between day 2 and baseline (day -1); ** indicates significance between day 7 and day 2.

Contrast enhancement within selected organs of each group is illustrated in Fig 3. There was no significant difference in mean HU or percent enhancement between groups in any organ (p = 0.6); however, a subjective difference in contrast enhancement was observed in all targeted organs. The subjective increase in contrast enhancement from baseline was observed in 3/6 dogs (1 dog from group 1 and 2 dogs from group 2).

When organ systems were compared over time, there was a significant increase in contrast enhancement within the liver, heart, aorta, and caudal vena cava at day 2 compared to baseline. Subjective contrast enhancement was noted within the spleen at this time; however, this finding was not significant. At day 7 p.i., contrast enhancement within the heart, aorta, and caudal vena cava was significantly decreased compared to day 2 p.i. whereas there was no difference within the liver and spleen. These values are summarized in Table 1.

**Table 1:** Mean contrast enhancement within select organ systems over time.

	Mean (HU)				
**Day**	Liver	Spleen	Heart	Aorta	Caudal vena cava
**-1**	62.92	67.07	46.93	41.95	44.17
**2**	78.52*	74.65	67.75*	62.48*	72.26*
**7**	73.68	69.01	44.67**	45.84**	38.67**

*indicates significance when value is compared to value at baseline (Day -1)

**indicates significance when value is compared to value Day 2

### Adverse events

The effects on heart rate, temperature, arterial blood pressure, and cardiac output are summarized in Table 2. All dogs had a normal heart rate and normal electrocardiogram with sinus rhythm. Two dogs exhibited mild hypotension and the remaining dogs were normotensive. One dog was from group 1 and had a mean systolic arterial pressure of 79.4 mmHg (range 66–89 mmHg) and mean arterial pressure of 53.8 mmHg (range 45–62 mmHg) throughout the infusion period. The second dog was from group 2 and had intermittent episodes of mild hypotension at 5, 20, 30, 35, and 45 minutes during infusion. There were no significant differences in arterial blood pressure, heart rate, body temperature, or cardiac output between groups or over time. No generalized external abnormalities, such as facial swelling, urticaria, wheals, or hyperemia were identified during CF800 infusion or throughout the study period. There were no clinically significant abnormalities in laboratory data.

**Table 2:** Summary of mean ± SD cardiovascular parameters for each group during Nanotrast-CF800 infusion.

Time (min)	Heart Rate (bpm)	Mean arterial pressure (mmHg)	Systolic arterial pressure (mmHg)	Diastolic arterial pressure (mmHg)	Cardiac output (L/min)
**Group 1**					
**0**	85.6 ± 5.1	73 ± 17.3	109.3 ± 26.5	57.6 ± 13.3	1.07 ± 0.17
**15**	73 ± 6.2	64.3 ± 7.0	92.3 ± 9.7	53 ± 7.9	0.99 ± 0.10
**30**	75.3 ± 8.6	73.3 ± 24.0	97.3 ± 18.15	51.6 ± 11.1	0.96 ± 0.15
**45**	81 ± 26.1	65 ± 12.3	94.6 ± 15.7	52 ± 9.6	0.88 ± 0.03
**Group 2**					
**0**	104.3 ± 11.7	70 ± 10.8	94.3 ± 13.5	58 ± 10.4	1.29 ± 0.28
**15**	92.6 ± 15.0	65.3 ± 8.4	97.3 ± 14.3	52.3 ± 6.8	1.07 ± 0.15
**30**	72 ± 15.5	60.3 ± 9.6	96 ± 13.7	47 ± 8.0	1.04 ± 0.36
**45**	76.6 ± 23.5	61 ± 5.3	97.6 ± 8.7	49.3 ± 6.6	1.04 ± 0.60
**60**	75.6 ± 19.6	62.3 ± 9.7	98.3 ± 11.8	48.6 ± 8.6	1.13 ± 0.61
**75**	82.3 ± 22.4	76 ± 30.4	112.6 ± 43.8	60.6 ± 25.6	1.02 ± 0.20

## Discussion

All dogs exhibited mild to moderate sedation and nausea during the test dose of CF800, which was short-lasting (several minutes) and spontaneously resolved without intervention. These clinical signs could have been associated with mild histamine release. To the authors’ knowledge, this is not a previously reported adverse effect of CF800 or liposomal nanoparticles since published studies have been performed on subjects anesthetized at the time of contrast administration [17, 18] or were not stated [12, 15]. If CF800 will be administered in the awake patient, administration of an anti-emetic medication could be considered to manage the effects of nausea.

The overall findings from this study demonstrate that the intravenous administration of CF800 was associated with no systemic adverse events in healthy dogs. Liposomes and lipid-based nanoparticles have been reported to stimulate the innate immunity through activation of the complement system. This subsequently induces inflammatory cells, such as, mast cells, basophils, and platelets, to release vasoactive mediators, including histamine [19, 20]. In humans and animal models, activation of complement by liposomes can affect the cardiovascular, respiratory, and cutaneous systems. In dogs, hemodynamic changes (tachy- and bradyarrhythmias, hypo- and hypertension), transient clinicopathologic abnormalities (leukopenia, thrombocytopenia), and occasional skin reactions have been reported [20]. Rarely, anaphylactic reactions have been reported in humans [21]. In preclinical studies in laboratory animals, no systemic adverse events were observed following intravenous injection of CF800 [12, 15, 17, 18, 22]. In our study, cardiovascular parameters were evaluated for any abnormal changes during contrast infusion; however, no significant changes were identified.

The histamine concentration was higher in group 2 dogs compared to group 1 dogs. The histamine concentration was demonstrated to peak at 30 minutes following CF800 infusion in both groups then sharply decline; however, no anticipated hypotension occurred during this time. Drug-induced histamine release and its effects on variable physiologic parameters has been evaluated in dogs receiving morphine [21, 23, 24] and muscle relaxants [24]. Studies in dogs receiving morphine reported variable histamine concentrations; however, dogs receiving a higher dose of morphine had significantly higher histamine release compared to histamine concentrations in our study. In addition, histamine release was associated with minimal cardiovascular abnormalities [21, 23]. To the authors’ knowledge, the evaluation of histamine release following the administration of liposomal nanoparticles has not been previously reported.

Only 2 dogs exhibited mild hypotension during infusion of CF800. The most common cardiovascular side effects associated with significant histamine release are vasodilation and tachycardia., In humans and dogs, normal plasma histamine concentration ranges between 0.2–1.4 ng/mL [25] and up to 0.8 ng/mL [21], respectively. Increased histamine concentrations were associated with dose-related hypotension following administration of muscle relaxants and morphine in dogs in one study [24]. In another study of dogs [21], maximal plasma histamine concentrations were 9.7 ng/mL and 589 ng/mL following high doses of hydromorphone (0.2 mg/kg IV) and morphine (1.0 mg/kg IV), respectively. All dogs remained normotensive throughout the study; however, 1 dog exhibited moderate hypotension corresponding with histamine concentrations of 589 ng/mL [21]. In our study, one dog in group 2 had a plasma histamine concentration of 44.08 ng/mL, which was the highest concentration in the study and was not associated with hypotension or any other cardiovascular side effects. The mild hypotension exhibited by the two dogs could be associated with isoflurane anesthesia and lack of stimulus. Thus, administration of CF800 with the doses used in our study did not appear to stimulate a high enough histamine release to cause significant changes in physiologic parameters in these dogs.

Interestingly, a positive relationship between histamine concentration and mean arterial pressure was identified. This finding is contradictory to previous studies in which an increasing plasma histamine concentration was associated with hypotension [21, 24]. However, in a study comparing plasma histamine levels in dogs receiving intravenous hydromorphone and morphine, significant cardiovascular effects were not detected despite varying concentrations of plasma histamine levels measured [21]. The authors of that study discussed that histamine can stimulate adrenal secretion of epinephrine and it has positive chronotropic and inotropic effects that increase cardiac output, which could potentially counteract its vasodilatory effects [21]. This positive chronotropic and inotropic effect is less likely in our study as sympathetic nervous response may be diminished in anesthetized dogs. It is possible that this finding could be due to low statistical power and further investigation in a larger sample size is warranted.

Iodinated contrast agents and ICG have small molecular weights and are rapidly distributed and excreted, which prevents the ability to perform longitudinal preoperative and intraoperative imaging studies and repeated contrast administration is necessary [26]. Liposomes have a larger molecular weight, which allows for prolonged retention within the vascular system and decreases its volume of distribution [26]. Published studies have consistently demonstrated the prolonged vascular half-life of liposome encapsulated contrast agents, including Nanotrast-CF800, in preclinical animal models ranging from 18–72 hours [12, 15, 17, 18, 22]. Vascular half-life of the contrast agent can be determined by measuring ROI within the aorta or left ventricle, which correlates with serological half-life [12, 15]. Taking this into account, the overall contrast enhancement within the aorta and left ventricle occurred at day 2 p.i. in our study, which is consistent with the results of previous studies [12, 15, 17].

Contrast enhancement was detected on CT images within the liver, heart, aorta, and caudal vena cava at 2 days p.i., which was significantly different from baseline. It is important to note that an increase in contrast enhancement from baseline was only observed in 3/6 dogs (1 dog from group 1 and 2 dogs from group 2). Since dogs were scanned at 2 days p.i., it is possible that peak contrast enhancement occurred earlier in these dogs and was missed. The dose administered in this study may still be too low of a dose for consistent uptake. Furthermore, contrast enhancement was still present within the liver at day 7 p.i. This is longer than previous studies, which report the visualization of contrast enhancement in preclinical animal model studies between 1 to 4 days p.i. of CF800 [12, 15, 17]. Contrast enhancement was significantly decreased within the heart, aorta, and caudal vena cava at day 7 p.i. compared to day 2 p.i. This could reflect the redistribution and/or excretion of the nanoparticle throughout the blood stream. These findings demonstrate that CF800 can accumulate within various organ systems, particularly the liver, allowing for prolonged retention of the agent and identification of contrast enhancement; however, studies to determine an appropriate dose or volume of CF800 that provides consistent contrast enhancement is warranted.

A limitation of this study included a small sample size, as these small numbers may affect the power for statistics demonstrated by the positive correlation between histamine and MAP. In addition, this study was performed on healthy research dogs, thus, pharmacokinetics of CF800 and the development of systemic adverse effects may differ in oncologic patients. Patients with cancer are typically middle to older aged animals and may have concurrent morbidities, which could affect the distribution and excretion of the contrast agent. Studies evaluating CF800 demonstrate CT contrast enhancement within the primary tumour between 24–96 hours p.i. [12, 15]. In our study, CTL was performed at 2 and 7 days p.i. Additional time points for CTL performed closer to the time of injection would be useful to better determine the earliest time to detection of contrast enhancement, as well as to confirm peak contrast enhancement. Based on peak mean plasma concentration of iohexol at 1 day p.i, it would have been interesting to correlate this finding with its contrast attenuation on CTL images. Clinically, the administration of CF800 and CT imaging can be performed in conscious or sedated dogs while dogs undergoing surgery will require general anesthesia. This study evaluated CF800 in anesthetized dogs, which may have masked any further clinical adverse events, as exhibited by the mild to moderate sedative and hypersalivation effects noted during the test dose. Future studies in which CF800 is administered should be performed in conscious clinically healthy dogs to further evaluate these effects. Pre-treatment with an anti-nausea medication and diphenhydramine immediately prior to CF800 administration should be considered.

## Conclusion

This study demonstrates that the intravenous administration of Nanotrast-CF800 was safe to use in healthy dogs at the reported doses. The higher dose should be considered as this may provide enhanced contrast images. Prolonged contrast enhancement was demonstrated within the liver at 7 days p.i.. While fluorescence is anticipated to parallel contrast enhancement [6], evaluation of fluorescence detection using NIRF was beyond the scope of this study and was not performed. Future investigations aim to utilize CF800 in the clinical setting with evaluation of CTL and NIRF imaging for cancer surgery dogs.

## Supporting information

S1 File Histamineeng_corrected. This is statistical documentation for histamine levels.
(DOCX)

S1 Table PK and histamine plasma concentrations. This is the data for measured anesthesia and lab parameters.
(XLSX)

S2 Table Anesthesia + lab parameters. This is the data for measured anesthesia and lab parameters.
(XLSX)
