# Supplementary material for: Canine preclinical safety evaluation of a multimodal nanoparticle agent (Nanotrast-CF800) for image-guided cancer surgery
Source: PLoS One. 2024 Jun 13;19(6):e0296913. doi: 10.1371/journal.pone.0296913 (PMC11175465; doi:10.1371/journal.pone.0296913)
Supplement: S1 File — This is statistical documentation for histamine levels. (DOCX) [file pone.0296913.s001.docx]

The SAS System

The Mixed Procedure

Model Information

Data Set WORK.HIST

Dependent Variable lhistamineng

Covariance Structure Autoregressive

Subject Effect id

Estimation Method REML

Residual Variance Method Profile

Fixed Effects SE Method Kenward-Roger

Degrees of Freedom Method Kenward-Roger

Dimensions

Covariance Parameters 2

Columns in X 27

Columns in Z 0

Subjects 6

Max Obs per Subject 9

Number of Observations

Number of Observations Read 45

Number of Observations Used 45

Number of Observations Not Used 0

Covariance Parameter Estimates

Standard Z

Cov Parm Subject Estimate Error Value Pr Z Alpha Lower Upper

AR(1) id 0.1789 0.1805 0.99 0.3216 0.05 -0.1748 0.5326

Residual 0.3007 0.07955 3.78 <.0001 0.05 0.1901 0.5462

Fit Statistics

-2 Res Log Likelihood 64.7

AIC (Smaller is Better) 68.7

AICC (Smaller is Better) 69.2

BIC (Smaller is Better) 68.3

Null Model Likelihood Ratio Test

DF Chi-Square Pr > ChiSq

1 0.96 0.3279

The SAS System

The Mixed Procedure

Solution for Fixed Effects

Standard

Effect group minutes Estimate Error DF t Value Pr > |t|

Intercept -0.3227 0.3166 28.6 -1.02 0.3166

group 1 -0.6103 0.4477 28.6 -1.36 0.1835

group 2 0 . . . .

minutes 0 0.1852 0.4477 28.6 0.41 0.6822

minutes 15 0.08141 0.4477 28.6 0.18 0.8570

minutes 30 2.5656 0.4478 28.6 5.73 <.0001

minutes 45 0.002278 0.4481 28.6 0.01 0.9960

minutes 60 -0.2288 0.4489 28.8 -0.51 0.6141

minutes 75 -0.1536 0.4506 29.3 -0.34 0.7356

minutes 90 -0.1353 0.4487 30 -0.30 0.7650

minutes 105 -0.3491 0.4083 21.5 -0.86 0.4020

minutes 120 0 . . . .

group*minutes 1 0 0.2570 0.6337 28.6 0.41 0.6881

group*minutes 1 15 0.06444 0.6349 28.8 0.10 0.9199

group*minutes 1 30 -0.8820 0.6372 29.3 -1.38 0.1768

group*minutes 1 45 -0.08770 0.6345 30 -0.14 0.8910

group*minutes 1 60 -0.1162 0.5774 21.5 -0.20 0.8424

group*minutes 1 75 0 . . . .

group*minutes 2 0 0 . . . .

group*minutes 2 15 0 . . . .

group*minutes 2 30 0 . . . .

group*minutes 2 45 0 . . . .

group*minutes 2 60 0 . . . .

group*minutes 2 75 0 . . . .

group*minutes 2 90 0 . . . .

group*minutes 2 105 0 . . . .

group*minutes 2 120 0 . . . .

Type 3 Tests of Fixed Effects

Num Den

Effect DF DF F Value Pr > F

group 1 10.2 12.95 0.0047

minutes 8 23.3 11.33 <.0001

group*minutes 5 23.3 0.81 0.5540

Least Squares Means

Standard

Effect group minutes Estimate Error DF t Value Pr > |t| Alpha

group 1 Non-est . . . . .

group 2 -0.1041 0.1188 9.32 -0.88 0.4031 0.05

The SAS System

The Mixed Procedure

Least Squares Means

Standard

Effect group minutes Estimate Error DF t Value Pr > |t| Alpha

minutes 0 -0.3141 0.2239 28.6 -1.40 0.1713 0.05

minutes 15 -0.5142 0.2239 28.6 -2.30 0.0291 0.05

minutes 30 1.4968 0.2239 28.6 6.69 <.0001 0.05

minutes 45 -0.6694 0.2239 28.6 -2.99 0.0057 0.05

minutes 60 -0.9148 0.2239 28.6 -4.09 0.0003 0.05

minutes 75 -0.7814 0.2239 28.6 -3.49 0.0016 0.05

minutes 90 Non-est . . . . .

minutes 105 Non-est . . . . .

minutes 120 Non-est . . . . .

group*minutes 1 0 -0.4908 0.3166 28.6 -1.55 0.1321 0.05

group*minutes 1 15 -0.7871 0.3166 28.6 -2.49 0.0190 0.05

group*minutes 1 30 0.7506 0.3166 28.6 2.37 0.0247 0.05

group*minutes 1 45 -1.0184 0.3166 28.6 -3.22 0.0032 0.05

group*minutes 1 60 -1.2781 0.3166 28.6 -4.04 0.0004 0.05

group*minutes 1 75 -1.0866 0.3166 28.6 -3.43 0.0018 0.05

group*minutes 2 0 -0.1375 0.3166 28.6 -0.43 0.6674 0.05

group*minutes 2 15 -0.2413 0.3166 28.6 -0.76 0.4522 0.05

group*minutes 2 30 2.2429 0.3166 28.6 7.08 <.0001 0.05

group*minutes 2 45 -0.3204 0.3166 28.6 -1.01 0.3200 0.05

group*minutes 2 60 -0.5515 0.3166 28.6 -1.74 0.0922 0.05

group*minutes 2 75 -0.4763 0.3166 28.6 -1.50 0.1434 0.05

group*minutes 2 90 -0.4580 0.3166 28.6 -1.45 0.1588 0.05

group*minutes 2 105 -0.6718 0.3166 28.6 -2.12 0.0426 0.05

group*minutes 2 120 -0.3227 0.3166 28.6 -1.02 0.3166 0.05

Least Squares Means

Effect group minutes Lower Upper

group 1 . .

group 2 -0.3715 0.1633

minutes 0 -0.7723 0.1440

minutes 15 -0.9724 -0.05608

minutes 30 1.0386 1.9549

minutes 45 -1.1276 -0.2113

minutes 60 -1.3729 -0.4567

minutes 75 -1.2396 -0.3233

minutes 90 . .

minutes 105 . .

minutes 120 . .

group*minutes 1 0 -1.1387 0.1571

group*minutes 1 15 -1.4351 -0.1392

group*minutes 1 30 0.1027 1.3985

group*minutes 1 45 -1.6663 -0.3705

group*minutes 1 60 -1.9260 -0.6302

group*minutes 1 75 -1.7345 -0.4387

The SAS System

The Mixed Procedure

Least Squares Means

Effect group minutes Lower Upper

group*minutes 2 0 -0.7854 0.5104

group*minutes 2 15 -0.8892 0.4066

group*minutes 2 30 1.5950 2.8908

group*minutes 2 45 -0.9683 0.3275

group*minutes 2 60 -1.1995 0.09636

group*minutes 2 75 -1.1242 0.1716

group*minutes 2 90 -1.1059 0.1899

group*minutes 2 105 -1.3197 -0.02387

group*minutes 2 120 -0.9706 0.3252

Differences of Least Squares Means

Standard

Effect group minutes _group _minutes Estimate Error DF t Value

group 1 2 Non-est . . .

minutes 0 15 0.2001 0.2887 21.5 0.69

minutes 0 30 -1.8109 0.3173 30 -5.71

minutes 0 45 0.3553 0.3186 29.3 1.12

minutes 0 60 0.6007 0.3174 28.8 1.89

minutes 0 75 0.4673 0.3169 28.6 1.47

minutes 0 90 Non-est . . .

minutes 0 105 Non-est . . .

minutes 0 120 Non-est . . .

minutes 15 30 -2.0110 0.2887 21.5 -6.97

minutes 15 45 0.1552 0.3173 30 0.49

minutes 15 60 0.4006 0.3186 29.3 1.26

Differences of Least Squares Means

Effect group minutes _group _minutes Pr > |t| Alpha Lower Upper

group 1 2 . . . .

minutes 0 15 0.4956 0.05 -0.3994 0.7996

minutes 0 30 <.0001 0.05 -2.4589 -1.1629

minutes 0 45 0.2738 0.05 -0.2960 1.0066

minutes 0 60 0.0686 0.05 -0.04880 1.2502

minutes 0 75 0.1512 0.05 -0.1811 1.1157

minutes 0 90 . . . .

minutes 0 105 . . . .

minutes 0 120 . . . .

minutes 15 30 <.0001 0.05 -2.6105 -1.4115

minutes 15 45 0.6283 0.05 -0.4928 0.8032

minutes 15 60 0.2186 0.05 -0.2507 1.0519

The SAS System

The Mixed Procedure

Differences of Least Squares Means

Standard

Effect group minutes _group _minutes Estimate Error DF t Value

minutes 15 75 0.2672 0.3174 28.8 0.84

minutes 15 90 Non-est . . .

minutes 15 105 Non-est . . .

minutes 15 120 Non-est . . .

minutes 30 45 2.1662 0.2887 21.5 7.50

minutes 30 60 2.4116 0.3173 30 7.60

minutes 30 75 2.2782 0.3186 29.3 7.15

minutes 30 90 Non-est . . .

minutes 30 105 Non-est . . .

minutes 30 120 Non-est . . .

minutes 45 60 0.2454 0.2887 21.5 0.85

minutes 45 75 0.1120 0.3173 30 0.35

minutes 45 90 Non-est . . .

minutes 45 105 Non-est . . .

minutes 45 120 Non-est . . .

minutes 60 75 -0.1334 0.2887 21.5 -0.46

minutes 60 90 Non-est . . .

minutes 60 105 Non-est . . .

minutes 60 120 Non-est . . .

minutes 75 90 Non-est . . .

minutes 75 105 Non-est . . .

minutes 75 120 Non-est . . .

minutes 90 105 0.2137 0.4083 21.5 0.52

minutes 90 120 -0.1353 0.4487 30 -0.30

minutes 105 120 -0.3491 0.4083 21.5 -0.86

group*minutes 1 0 1 15 0.2964 0.4083 21.5 0.73

group*minutes 1 0 1 30 -1.2414 0.4487 30 -2.77

group*minutes 1 0 1 45 0.5277 0.4506 29.3 1.17

group*minutes 1 0 1 60 0.7873 0.4489 28.8 1.75

group*minutes 1 0 1 75 0.5958 0.4481 28.6 1.33

group*minutes 1 0 2 0 -0.3533 0.4477 28.6 -0.79

group*minutes 1 0 2 15 -0.2495 0.4477 28.6 -0.56

group*minutes 1 0 2 30 -2.7337 0.4477 28.6 -6.11

group*minutes 1 0 2 45 -0.1703 0.4477 28.6 -0.38

group*minutes 1 0 2 60 0.06078 0.4477 28.6 0.14

group*minutes 1 0 2 75 -0.01449 0.4477 28.6 -0.03

group*minutes 1 0 2 90 -0.03274 0.4477 28.6 -0.07

group*minutes 1 0 2 105 0.1810 0.4477 28.6 0.40

group*minutes 1 0 2 120 -0.1681 0.4477 28.6 -0.38

group*minutes 1 15 1 30 -1.5378 0.4083 21.5 -3.77

group*minutes 1 15 1 45 0.2313 0.4487 30 0.52

group*minutes 1 15 1 60 0.4909 0.4506 29.3 1.09

group*minutes 1 15 1 75 0.2994 0.4489 28.8 0.67

group*minutes 1 15 2 0 -0.6497 0.4477 28.6 -1.45

group*minutes 1 15 2 15 -0.5459 0.4477 28.6 -1.22

group*minutes 1 15 2 30 -3.0301 0.4477 28.6 -6.77

The SAS System

The Mixed Procedure

Differences of Least Squares Means

Effect group minutes _group _minutes Pr > |t| Alpha Lower Upper

minutes 15 75 0.4069 0.05 -0.3823 0.9167

minutes 15 90 . . . .

minutes 15 105 . . . .

minutes 15 120 . . . .

minutes 30 45 <.0001 0.05 1.5667 2.7657

minutes 30 60 <.0001 0.05 1.7636 3.0596

minutes 30 75 <.0001 0.05 1.6269 2.9295

minutes 30 90 . . . .

minutes 30 105 . . . .

minutes 30 120 . . . .

minutes 45 60 0.4047 0.05 -0.3541 0.8449

minutes 45 75 0.7265 0.05 -0.5360 0.7600

minutes 45 90 . . . .

minutes 45 105 . . . .

minutes 45 120 . . . .

minutes 60 75 0.6487 0.05 -0.7328 0.4661

minutes 60 90 . . . .

minutes 60 105 . . . .

minutes 60 120 . . . .

minutes 75 90 . . . .

minutes 75 105 . . . .

minutes 75 120 . . . .

minutes 90 105 0.6059 0.05 -0.6340 1.0615

minutes 90 120 0.7650 0.05 -1.0517 0.7810

minutes 105 120 0.4020 0.05 -1.1968 0.4987

group*minutes 1 0 1 15 0.4757 0.05 -0.5514 1.1441

group*minutes 1 0 1 30 0.0096 0.05 -2.1578 -0.3251

group*minutes 1 0 1 45 0.2510 0.05 -0.3935 1.4488

group*minutes 1 0 1 60 0.0901 0.05 -0.1312 1.7058

group*minutes 1 0 1 75 0.1941 0.05 -0.3212 1.5128

group*minutes 1 0 2 0 0.4365 0.05 -1.2696 0.5630

group*minutes 1 0 2 15 0.5817 0.05 -1.1658 0.6668

group*minutes 1 0 2 30 <.0001 0.05 -3.6500 -1.8174

group*minutes 1 0 2 45 0.7064 0.05 -1.0866 0.7459

group*minutes 1 0 2 60 0.8930 0.05 -0.8555 0.9771

group*minutes 1 0 2 75 0.9744 0.05 -0.9308 0.9018

group*minutes 1 0 2 90 0.9422 0.05 -0.9490 0.8835

group*minutes 1 0 2 105 0.6890 0.05 -0.7353 1.0973

group*minutes 1 0 2 120 0.7101 0.05 -1.0843 0.7482

group*minutes 1 15 1 30 0.0011 0.05 -2.3856 -0.6900

group*minutes 1 15 1 45 0.6100 0.05 -0.6851 1.1476

group*minutes 1 15 1 60 0.2848 0.05 -0.4302 1.4120

group*minutes 1 15 1 75 0.5101 0.05 -0.6191 1.2179

group*minutes 1 15 2 0 0.1576 0.05 -1.5660 0.2666

group*minutes 1 15 2 15 0.2327 0.05 -1.4621 0.3704

group*minutes 1 15 2 30 <.0001 0.05 -3.9463 -2.1138

The SAS System

The Mixed Procedure

Differences of Least Squares Means

Standard

Effect group minutes _group _minutes Estimate Error DF t Value

group*minutes 1 15 2 45 -0.4667 0.4477 28.6 -1.04

group*minutes 1 15 2 60 -0.2356 0.4477 28.6 -0.53

group*minutes 1 15 2 75 -0.3109 0.4477 28.6 -0.69

group*minutes 1 15 2 90 -0.3291 0.4477 28.6 -0.74

group*minutes 1 15 2 105 -0.1154 0.4477 28.6 -0.26

group*minutes 1 15 2 120 -0.4644 0.4477 28.6 -1.04

group*minutes 1 30 1 45 1.7691 0.4083 21.5 4.33

group*minutes 1 30 1 60 2.0287 0.4487 30 4.52

group*minutes 1 30 1 75 1.8372 0.4506 29.3 4.08

group*minutes 1 30 2 0 0.8881 0.4477 28.6 1.98

group*minutes 1 30 2 15 0.9919 0.4477 28.6 2.22

group*minutes 1 30 2 30 -1.4923 0.4477 28.6 -3.33

group*minutes 1 30 2 45 1.0711 0.4477 28.6 2.39

group*minutes 1 30 2 60 1.3022 0.4477 28.6 2.91

group*minutes 1 30 2 75 1.2269 0.4477 28.6 2.74

group*minutes 1 30 2 90 1.2087 0.4477 28.6 2.70

group*minutes 1 30 2 105 1.4224 0.4477 28.6 3.18

group*minutes 1 30 2 120 1.0733 0.4477 28.6 2.40

group*minutes 1 45 1 60 0.2596 0.4083 21.5 0.64

group*minutes 1 45 1 75 0.06815 0.4487 30 0.15

group*minutes 1 45 2 0 -0.8810 0.4477 28.6 -1.97

group*minutes 1 45 2 15 -0.7771 0.4477 28.6 -1.74

group*minutes 1 45 2 30 -3.2613 0.4477 28.6 -7.28

group*minutes 1 45 2 45 -0.6980 0.4477 28.6 -1.56

group*minutes 1 45 2 60 -0.4669 0.4477 28.6 -1.04

group*minutes 1 45 2 75 -0.5421 0.4477 28.6 -1.21

group*minutes 1 45 2 90 -0.5604 0.4477 28.6 -1.25

group*minutes 1 45 2 105 -0.3466 0.4477 28.6 -0.77

group*minutes 1 45 2 120 -0.6957 0.4477 28.6 -1.55

group*minutes 1 60 1 75 -0.1915 0.4083 21.5 -0.47

group*minutes 1 60 2 0 -1.1406 0.4477 28.6 -2.55

group*minutes 1 60 2 15 -1.0368 0.4477 28.6 -2.32

group*minutes 1 60 2 30 -3.5210 0.4477 28.6 -7.86

group*minutes 1 60 2 45 -0.9576 0.4477 28.6 -2.14

group*minutes 1 60 2 60 -0.7265 0.4477 28.6 -1.62

group*minutes 1 60 2 75 -0.8018 0.4477 28.6 -1.79

group*minutes 1 60 2 90 -0.8200 0.4477 28.6 -1.83

group*minutes 1 60 2 105 -0.6063 0.4477 28.6 -1.35

group*minutes 1 60 2 120 -0.9554 0.4477 28.6 -2.13

group*minutes 1 75 2 0 -0.9491 0.4477 28.6 -2.12

group*minutes 1 75 2 15 -0.8453 0.4477 28.6 -1.89

group*minutes 1 75 2 30 -3.3295 0.4477 28.6 -7.44

group*minutes 1 75 2 45 -0.7662 0.4477 28.6 -1.71

group*minutes 1 75 2 60 -0.5350 0.4477 28.6 -1.20

group*minutes 1 75 2 75 -0.6103 0.4477 28.6 -1.36

group*minutes 1 75 2 90 -0.6285 0.4477 28.6 -1.40

The SAS System

The Mixed Procedure

Differences of Least Squares Means

Effect group minutes _group _minutes Pr > |t| Alpha Lower Upper

group*minutes 1 15 2 45 0.3059 0.05 -1.3830 0.4496

group*minutes 1 15 2 60 0.6028 0.05 -1.1519 0.6807

group*minutes 1 15 2 75 0.4931 0.05 -1.2271 0.6054

group*minutes 1 15 2 90 0.4683 0.05 -1.2454 0.5872

group*minutes 1 15 2 105 0.7985 0.05 -1.0316 0.8009

group*minutes 1 15 2 120 0.3083 0.05 -1.3807 0.4518

group*minutes 1 30 1 45 0.0003 0.05 0.9213 2.6168

group*minutes 1 30 1 60 <.0001 0.05 1.1123 2.9451

group*minutes 1 30 1 75 0.0003 0.05 0.9161 2.7583

group*minutes 1 30 2 0 0.0570 0.05 -0.02817 1.8044

group*minutes 1 30 2 15 0.0349 0.05 0.07566 1.9082

group*minutes 1 30 2 30 0.0024 0.05 -2.4086 -0.5760

group*minutes 1 30 2 45 0.0236 0.05 0.1548 1.9873

group*minutes 1 30 2 60 0.0070 0.05 0.3859 2.2185

group*minutes 1 30 2 75 0.0105 0.05 0.3106 2.1432

group*minutes 1 30 2 90 0.0115 0.05 0.2924 2.1250

group*minutes 1 30 2 105 0.0036 0.05 0.5061 2.3387

group*minutes 1 30 2 120 0.0233 0.05 0.1571 1.9896

group*minutes 1 45 1 60 0.5315 0.05 -0.5881 1.1074

group*minutes 1 45 1 75 0.8803 0.05 -0.8482 0.9845

group*minutes 1 45 2 0 0.0589 0.05 -1.7972 0.03531

group*minutes 1 45 2 15 0.0934 0.05 -1.6934 0.1391

group*minutes 1 45 2 30 <.0001 0.05 -4.1776 -2.3451

group*minutes 1 45 2 45 0.1300 0.05 -1.6143 0.2183

group*minutes 1 45 2 60 0.3058 0.05 -1.3832 0.4494

group*minutes 1 45 2 75 0.2358 0.05 -1.4584 0.3741

group*minutes 1 45 2 90 0.2208 0.05 -1.4767 0.3559

group*minutes 1 45 2 105 0.4451 0.05 -1.2629 0.5696

group*minutes 1 45 2 120 0.1312 0.05 -1.6120 0.2206

group*minutes 1 60 1 75 0.6438 0.05 -1.0393 0.6563

group*minutes 1 60 2 0 0.0165 0.05 -2.0569 -0.2243

group*minutes 1 60 2 15 0.0280 0.05 -1.9531 -0.1205

group*minutes 1 60 2 30 <.0001 0.05 -4.4373 -2.6047

group*minutes 1 60 2 45 0.0411 0.05 -1.8739 -0.04137

group*minutes 1 60 2 60 0.1156 0.05 -1.6428 0.1898

group*minutes 1 60 2 75 0.0839 0.05 -1.7181 0.1145

group*minutes 1 60 2 90 0.0775 0.05 -1.7363 0.09624

group*minutes 1 60 2 105 0.1863 0.05 -1.5226 0.3100

group*minutes 1 60 2 120 0.0416 0.05 -1.8716 -0.03909

group*minutes 1 75 2 0 0.0428 0.05 -1.8654 -0.03284

group*minutes 1 75 2 15 0.0692 0.05 -1.7616 0.07099

group*minutes 1 75 2 30 <.0001 0.05 -4.2458 -2.4132

group*minutes 1 75 2 45 0.0979 0.05 -1.6824 0.1501

group*minutes 1 75 2 60 0.2419 0.05 -1.4513 0.3812

group*minutes 1 75 2 75 0.1835 0.05 -1.5266 0.3060

group*minutes 1 75 2 90 0.1711 0.05 -1.5448 0.2877

The SAS System

The Mixed Procedure

Differences of Least Squares Means

Standard

Effect group minutes _group _minutes Estimate Error DF t Value

group*minutes 1 75 2 105 -0.4148 0.4477 28.6 -0.93

group*minutes 1 75 2 120 -0.7639 0.4477 28.6 -1.71

group*minutes 2 0 2 15 0.1038 0.4083 21.5 0.25

group*minutes 2 0 2 30 -2.3804 0.4487 30 -5.31

group*minutes 2 0 2 45 0.1830 0.4506 29.3 0.41

group*minutes 2 0 2 60 0.4141 0.4489 28.8 0.92

group*minutes 2 0 2 75 0.3388 0.4481 28.6 0.76

group*minutes 2 0 2 90 0.3206 0.4478 28.6 0.72

group*minutes 2 0 2 105 0.5343 0.4477 28.6 1.19

group*minutes 2 0 2 120 0.1852 0.4477 28.6 0.41

group*minutes 2 15 2 30 -2.4842 0.4083 21.5 -6.08

group*minutes 2 15 2 45 0.07913 0.4487 30 0.18

group*minutes 2 15 2 60 0.3103 0.4506 29.3 0.69

group*minutes 2 15 2 75 0.2350 0.4489 28.8 0.52

group*minutes 2 15 2 90 0.2167 0.4481 28.6 0.48

group*minutes 2 15 2 105 0.4305 0.4478 28.6 0.96

group*minutes 2 15 2 120 0.08141 0.4477 28.6 0.18

group*minutes 2 30 2 45 2.5633 0.4083 21.5 6.28

group*minutes 2 30 2 60 2.7945 0.4487 30 6.23

group*minutes 2 30 2 75 2.7192 0.4506 29.3 6.04

group*minutes 2 30 2 90 2.7010 0.4489 28.8 6.02

group*minutes 2 30 2 105 2.9147 0.4481 28.6 6.50

group*minutes 2 30 2 120 2.5656 0.4478 28.6 5.73

group*minutes 2 45 2 60 0.2311 0.4083 21.5 0.57

group*minutes 2 45 2 75 0.1559 0.4487 30 0.35

group*minutes 2 45 2 90 0.1376 0.4506 29.3 0.31

group*minutes 2 45 2 105 0.3514 0.4489 28.8 0.78

group*minutes 2 45 2 120 0.002278 0.4481 28.6 0.01

group*minutes 2 60 2 75 -0.07527 0.4083 21.5 -0.18

group*minutes 2 60 2 90 -0.09352 0.4487 30 -0.21

group*minutes 2 60 2 105 0.1202 0.4506 29.3 0.27

group*minutes 2 60 2 120 -0.2288 0.4489 28.8 -0.51

group*minutes 2 75 2 90 -0.01825 0.4083 21.5 -0.04

group*minutes 2 75 2 105 0.1955 0.4487 30 0.44

group*minutes 2 75 2 120 -0.1536 0.4506 29.3 -0.34

group*minutes 2 90 2 105 0.2137 0.4083 21.5 0.52

group*minutes 2 90 2 120 -0.1353 0.4487 30 -0.30

group*minutes 2 105 2 120 -0.3491 0.4083 21.5 -0.86

Differences of Least Squares Means

Effect group minutes _group _minutes Pr > |t| Alpha Lower Upper

group*minutes 1 75 2 105 0.3620 0.05 -1.3311 0.5015

group*minutes 1 75 2 120 0.0988 0.05 -1.6802 0.1524

The SAS System

The Mixed Procedure

Differences of Least Squares Means

Effect group minutes _group _minutes Pr > |t| Alpha Lower Upper

group*minutes 2 0 2 15 0.8017 0.05 -0.7439 0.9516

group*minutes 2 0 2 30 <.0001 0.05 -3.2967 -1.4640

group*minutes 2 0 2 45 0.6876 0.05 -0.7381 1.1041

group*minutes 2 0 2 60 0.3640 0.05 -0.5044 1.3326

group*minutes 2 0 2 75 0.4558 0.05 -0.5782 1.2558

group*minutes 2 0 2 90 0.4799 0.05 -0.5959 1.2371

group*minutes 2 0 2 105 0.2425 0.05 -0.3820 1.4506

group*minutes 2 0 2 120 0.6822 0.05 -0.7311 1.1015

group*minutes 2 15 2 30 <.0001 0.05 -3.3320 -1.6364

group*minutes 2 15 2 45 0.8612 0.05 -0.8372 0.9955

group*minutes 2 15 2 60 0.4965 0.05 -0.6109 1.2314

group*minutes 2 15 2 75 0.6047 0.05 -0.6835 1.1535

group*minutes 2 15 2 90 0.6323 0.05 -0.7003 1.1338

group*minutes 2 15 2 105 0.3445 0.05 -0.4860 1.3470

group*minutes 2 15 2 120 0.8570 0.05 -0.8349 0.9977

group*minutes 2 30 2 45 <.0001 0.05 1.7156 3.4111

group*minutes 2 30 2 60 <.0001 0.05 1.8781 3.7108

group*minutes 2 30 2 75 <.0001 0.05 1.7981 3.6403

group*minutes 2 30 2 90 <.0001 0.05 1.7824 3.6195

group*minutes 2 30 2 105 <.0001 0.05 1.9977 3.8317

group*minutes 2 30 2 120 <.0001 0.05 1.6491 3.4821

group*minutes 2 45 2 60 0.5772 0.05 -0.6166 1.0789

group*minutes 2 45 2 75 0.7307 0.05 -0.7605 1.0722

group*minutes 2 45 2 90 0.7622 0.05 -0.7835 1.0587

group*minutes 2 45 2 105 0.4402 0.05 -0.5672 1.2699

group*minutes 2 45 2 120 0.9960 0.05 -0.9147 0.9193

group*minutes 2 60 2 75 0.8555 0.05 -0.9230 0.7725

group*minutes 2 60 2 90 0.8363 0.05 -1.0099 0.8228

group*minutes 2 60 2 105 0.7915 0.05 -0.8009 1.0413

group*minutes 2 60 2 120 0.6141 0.05 -1.1474 0.6897

group*minutes 2 75 2 90 0.9648 0.05 -0.8660 0.8295

group*minutes 2 75 2 105 0.6662 0.05 -0.7209 1.1119

group*minutes 2 75 2 120 0.7356 0.05 -1.0747 0.7675

group*minutes 2 90 2 105 0.6059 0.05 -0.6340 1.0615

group*minutes 2 90 2 120 0.7650 0.05 -1.0517 0.7810

group*minutes 2 105 2 120 0.4020 0.05 -1.1968 0.4987

The SAS System

group minutes LL mean UL

1 _ . . .

2 _ 0.68973 0.90117 1.1774

_ 0 0.46197 0.73043 1.1549

_ 15 0.37819 0.59797 0.9455

_ 30 2.82538 4.46729 7.0634

_ 45 0.32382 0.51200 0.8095

_ 60 0.25336 0.40059 0.6334

_ 75 0.28951 0.45775 0.7238

_ 90 . . .

_ 105 . . .

_ 120 . . .

1 0 0.32024 0.61215 1.1702

1 15 0.23810 0.45514 0.8700

1 30 1.10820 2.11836 4.0493

1 45 0.18894 0.36116 0.6904

1 60 0.14573 0.27858 0.5325

1 75 0.17649 0.33737 0.6449

2 0 0.45595 0.87157 1.6660

2 15 0.41099 0.78561 1.5017

2 30 4.92841 9.42082 18.0082

2 45 0.37972 0.72584 1.3875

2 60 0.30136 0.57606 1.1012

2 75 0.32492 0.62109 1.1872

2 90 0.33090 0.63253 1.2091

2 105 0.26722 0.51080 0.9764

2 120 0.37885 0.72419 1.3843

The SAS System

The UNIVARIATE Procedure

Variable: Resid

Moments

N 45 Sum Weights 45

Mean 0 Sum Observations 0

Std Deviation 0.45361464 Variance 0.20576624

Skewness 0.25813825 Kurtosis 3.43431284

Uncorrected SS 9.05371451 Corrected SS 9.05371451

Coeff Variation . Std Error Mean 0.06762088

Basic Statistical Measures

Location Variability

Mean 0.00000 Std Deviation 0.45361

Median -0.01631 Variance 0.20577

Mode . Range 2.93674

Interquartile Range 0.44997

Tests for Location: Mu0=0

Test -Statistic- -----p Value------

Student's t t 0 Pr > |t| 1.0000

Sign M -1.5 Pr >= |M| 0.7660

Signed Rank S -18.5 Pr >= |S| 0.8373

Tests for Normality

Test --Statistic--- -----p Value------

Shapiro-Wilk W 0.943038 Pr < W 0.0278

Kolmogorov-Smirnov D 0.103816 Pr > D >0.1500

Cramer-von Mises W-Sq 0.097648 Pr > W-Sq 0.1203

Anderson-Darling A-Sq 0.678323 Pr > A-Sq 0.0755

Quantiles (Definition 5)

Level Quantile

100% Max 1.5149329

99% 1.5149329

95% 0.6549065

90% 0.4572095

75% Q3 0.2099050

50% Median -0.0163133

25% Q1 -0.2400639

The SAS System

The UNIVARIATE Procedure

Variable: Resid

Quantiles (Definition 5)

Level Quantile

10% -0.4585243

5% -0.5623743

1% -1.4218106

0% Min -1.4218106

Extreme Observations

------Lowest------ ------Highest-----

Value Obs Value Obs

-1.421811 9 0.256415 5

-0.686326 23 0.262241 30

-0.562374 19 0.387052 35

-0.537656 20 0.402048 34

-0.458524 22 0.425462 38

-0.401565 43 0.457210 33

-0.381430 39 0.648927 31

-0.335509 45 0.654906 32

-0.317302 24 0.802438 28

-0.252884 44 1.514933 3

Stem Leaf # Boxplot

14 1 1 0

12

10

8 0 1 |

6 55 2 |

4 036 3 |

2 14669 5 +-----+

0 378801238 9 | + |

-0 9843964420 10 *-----*

-2 84255420 8 +-----+

-4 6460 4 |

-6 9 1 |

-8

-10

-12

-14 2 1 0

----+----+----+----+

Multiply Stem.Leaf by 10**-1

The SAS System

The UNIVARIATE Procedure

Variable: Resid

Normal Probability Plot

1.5+ *

|

| +++

0.9+ *++++

| *+*+

| ++***

0.3+ ++****

| +******

| *******

-0.3+ ******+

| * ***+++

| *++++

-0.9+ +++++

|+++

|

-1.5+ *

+----+----+----+----+----+----+----+----+----+----+

-2 -1 0 +1 +2

The SAS System

Plot of Resid*Pred. Legend: A = 1 obs, B = 2 obs, etc.

Resid ‚

1.5 ˆ A

‚

‚

‚

‚

‚

‚

1.0 ˆ

‚

‚

‚ A

‚

‚ A A

‚

0.5 ˆ

‚ AA A

‚ A

‚ A A

‚ A A A

‚ AA A

‚ B A A

0.0 ˆ A A A A

‚ A A A

‚ A A

‚ A A A A A

‚ A B

‚ A A

‚ A A

-0.5 ˆ

‚ A A

‚

‚ A

‚

‚

‚

-1.0 ˆ

‚

‚

‚

‚

‚

‚ A

-1.5 ˆ

‚

Šƒˆƒƒƒƒƒƒƒƒƒˆƒƒƒƒƒƒƒƒƒˆƒƒƒƒƒƒƒƒƒˆƒƒƒƒƒƒƒƒƒˆƒƒƒƒƒƒƒƒƒˆƒƒƒƒƒƒƒƒƒˆƒƒƒƒƒƒƒƒƒˆƒƒƒƒƒƒƒƒƒˆƒ

-1.5 -1.0 -0.5 0.0 0.5 1.0 1.5 2.0 2.5

Pred

The SAS System

Plot of Resid*id. Legend: A = 1 obs, B = 2 obs, etc.

Resid ‚

1.5 ˆ A

‚

‚

‚

‚

‚

‚

1.0 ˆ

‚

‚

‚ A

‚

‚ B

‚

0.5 ˆ

‚ B A

‚ A

‚ A A

‚ A A A

‚ B A

‚ A A B

0.0 ˆ B A A

‚ A B

‚ A A

‚ B A B

‚ A A A

‚ B

‚ A A

-0.5 ˆ

‚ B

‚

‚ A

‚

‚

‚

-1.0 ˆ

‚

‚

‚

‚

‚

‚ A

-1.5 ˆ

‚

Šƒƒˆƒƒƒƒƒƒƒƒƒƒƒƒˆƒƒƒƒƒƒƒƒƒƒƒƒˆƒƒƒƒƒƒƒƒƒƒƒƒˆƒƒƒƒƒƒƒƒƒƒƒƒˆƒƒƒƒƒƒƒƒƒƒƒƒˆƒƒ

1 2 3 4 5 6

id

The SAS System

Plot of Resid*group. Legend: A = 1 obs, B = 2 obs, etc.

Resid ‚

1.5 ˆ A

‚

‚

‚

‚

‚

‚

1.0 ˆ

‚

‚

‚ A

‚

‚ B

‚

0.5 ˆ

‚ C

‚ A

‚ A A

‚ B A

‚ C

‚ D

0.0 ˆ B B

‚ C

‚ B

‚ C B

‚ A B

‚ B

‚ B

-0.5 ˆ

‚ B

‚

‚ A

‚

‚

‚

-1.0 ˆ

‚

‚

‚

‚

‚

‚ A

-1.5 ˆ

‚

Šƒƒˆƒƒƒƒƒƒƒƒƒƒƒƒƒƒƒƒƒƒƒƒƒƒƒƒƒƒƒƒƒƒƒƒƒƒƒƒƒƒƒƒƒƒƒƒƒƒƒƒƒƒƒƒƒƒˆƒƒ

1 2

group

The SAS System

Plot of Resid*minutes. Legend: A = 1 obs, B = 2 obs, etc.

Resid ‚

1.5 ˆ A

‚

‚

‚

‚

‚

‚

1.0 ˆ

‚

‚

‚ A

‚

‚ A A

‚

0.5 ˆ

‚ A A A

‚ A

‚ A A

‚ A A A

‚ A A A

‚ A A B

0.0 ˆ A B A

‚ A A A

‚ A A

‚ A B A A

‚ A A A

‚ A A

‚ A A

-0.5 ˆ

‚ A A

‚

‚ A

‚

‚

‚

-1.0 ˆ

‚

‚

‚

‚

‚

‚ A

-1.5 ˆ

‚

Šƒˆƒƒƒƒƒƒƒƒƒˆƒƒƒƒƒƒƒƒƒˆƒƒƒƒƒƒƒƒƒˆƒƒƒƒƒƒƒƒƒˆƒƒƒƒƒƒƒƒƒˆƒƒƒƒƒƒƒƒƒˆƒƒƒƒƒƒƒƒƒˆƒƒƒƒƒƒƒƒƒˆƒ

0 15 30 45 60 75 90 105 120

minutes

The SAS System

Plot of histamine*map. Symbol is value of group.

histamine ‚

120 ˆ

‚

‚

‚ 2

‚

‚

‚

100 ˆ

‚

‚2

‚

‚

‚ 1

‚

80 ˆ

‚

‚

‚

‚

‚

‚

60 ˆ

‚ 2

‚

‚

‚

‚

‚

40 ˆ

‚

‚

‚

‚

‚

‚

20 ˆ

‚ 1 2

‚

‚ 2 2

‚ 2 2 2

‚1 22 11 2 1

‚ 1 2 22 1 21 1 1 1

0 ˆ

‚

Šˆƒƒƒƒƒƒƒƒƒƒˆƒƒƒƒƒƒƒƒƒƒˆƒƒƒƒƒƒƒƒƒƒˆƒƒƒƒƒƒƒƒƒƒˆƒƒƒƒƒƒƒƒƒƒˆƒƒƒƒƒƒƒƒƒƒˆƒƒƒƒƒƒƒƒƒƒˆƒ

50 60 70 80 90 100 110 120

map

NOTE: 15 obs had missing values. 2 obs hidden.
